# Limited Renal Intravascular Lymphoma: A Case Report and Review of the Literature

**DOI:** 10.1155/2020/7052536

**Published:** 2020-10-06

**Authors:** Guillermo Enrique Quintero Vega, Daniel Osorio, José Antonio de la Hoz Valle, Daniela Rodríguez Feria

**Affiliations:** ^1^Department of Hematology, Hospital Universitario Fundación Santa Fe de Bogotá, Bogotá, Colombia; ^2^Department of Hematology, Universidad de los Andes, Bogotá, Colombia; ^3^Department of Clinical Research, Hospital Universitario Fundación Santa Fe de Bogotá, Bogotá, Colombia

## Abstract

Intravascular large B-cell lymphoma (IVLBCL) is a rare subtype of non-Hodgkin lymphoma. It is characterized by the proliferation of cancerous cells into the intraluminal space of the blood vessels. It has a low incidence rate of 0.095 cases per 1,000,000. The clinical presentation is insidious and unspecific, often delaying the diagnosis. IVLBCL can be diagnosed through body images and histopathology analysis. This neoplasm averages a 60% response rate to current chemotherapy treatment, favoring rituximab, and doxorubicin-based regimen if it is diagnosed in time. Here, we present the case of a 56-year-old man admitted to our hospital with a fever who was eventually diagnosed with IVLBCL. He presented to the consultation with anemia, fever, and splenomegaly. An infection panel, a bone marrow biopsy, and a PET-CT scan were performed and ruled out the possibility of infections and neoplasms. The patient later developed edematous syndrome. As a result, a renal biopsy was performed which tested positive for intravascular large B-cell lymphoma. Currently, the patient has been in complete remission for 33 months. Along with presenting this specific case, we also reviewed previously published cases of IVLBCL to illustrate the renal involvement of this pathology.

## 1. Introduction

Intravascular large B-cell lymphoma (IVLBCL) is a rare subtype of large cell lymphoma that is characterized by the proliferation of cancerous cells into the intraluminal space of small to medium blood vessels while sparing the surrounding tissue, lymph nodes, and reticuloendothelial system. IVLBCL's incidence rate is 0.095 cases per 1,000,000 in the United States [1]. IVLBCL patients have a median age at diagnosis of 70 years (the age range is from 40 to 90 years old). IVLBCL affects both males and females without a predilection for one or the other [2]. Also, IVLBCL has a heterogeneous clinical presentation depending on the organ that is compromised. The majority of patients present with systemic symptoms such as fever of an unknown origin, pain, weight loss, and rapid deterioration [3]. Usually, the central nervous system (CNS) and the skin are commonly affected [4]. This pathology is classified into three main variants: classical, cutaneous, and hemophagocytic [5].

A diagnosis is made by conducting a surgical biopsy in the affected organ to determine if there has been infiltration by cancerous cells. Intravascular lymphoma (IVL) is positive in 91% of cases for B-cell phenotype (CD19, CD20, CD22, and CD79a) and has occasionally shown expression of CD5 with this last marker being associated with a poor prognosis [6]. Vieites et al. and Yamamoto et al. [7, 8] described segmental tandem triplication of the 18q21 and q22q25 of chromosome 11 as genetic alterations of IVL.

The treatment consists of anthracycline-based chemotherapy regimens which have a nearly 60% response rate and a 3-year overall survival rate higher than 30% [3]. CNS-directed therapy, such as rituximab-cyclophosphamide, vincristine, and doxorubicin, is recommended for patients with neurologic symptoms. Prednisone regimens do not penetrate the CNS [4]. However, this information comes from a small series of cases, and given the rare nature of this disease, there are no randomized controlled trials that compare these treatment regimens [9]. The analysis of a small sample size of patients favors rituximab and doxorubicin-based treatments when adjusting for age and poor prognosis factors such as elevated lactate dehydrogenase serum levels [10]. Notably, patterns of recurrence seem to indicate that the same organ becomes compromised if it recurs. However, the small number of case reports available is not enough for statistical analysis [10].

## 2. Case Report

In 2017, a 56-year-old man arrived at the emergency room with fever, chills, dyspnea, arthralgia, and headache that he had been experiencing for 20 days before the medical consultation. He had mild anemia with a hemoglobin level of 9.7 g/dL (reference range (RR): 14-18 g/dL), lactate dehydrogenase with levels of 506 U/L (RR: 98-192 U/L), C-reactive protein levels of 18,904 mg/dL (RR: 0.000-0.748 mg/dL), beta 2 microglobulin levels of 5620.0 ng/mL (RR: 1000.0-3000.0 ng/mL), and albumin levels of 1.8 g/dL (RR: 3.50-4.80 g/dL). A physical examination showed fever, diaphoresis, tachypnea, blood oxygen saturation level of 76%, lung noises, and a palpable spleen one centimeter below the costal margin.

Because the patient presented with a fever, infectious disease panels were performed including IgM and IgG antibodies against Cytomegalovirus, rubella virus infection, Treponema pallidum, anti-Epstein-Barr virus-IgG and IgM, hepatitis B and C, and tropical diseases (thick drop test). He was also tested for central nervous system infections using cerebrospinal fluid analysis. All panels and tests were negative.

The patient also presented anemia and a high level of lactate dehydrogenase; therefore, a hematological malignancy was suspected. A bone marrow biopsy was conducted. The bone marrow biopsy tested negative for hemophagocytosis. However, it showed grade II fibrosis. Therefore, a second bone marrow biopsy was performed, which did not show fibrosis; thus, the fibrosis from the first biopsy was interpreted as an inflammatory condition. Also, an autoimmune panel (Anas, rheumatoid factor, C3 and C4 levels, anti-DNA, and anti-neutrophil cytoplasmic antibody) was performed. The autoimmune panel was also negative.

Additionally, bone marrow cultures for detecting bacterial and fungal infections were performed. Cultures for fungal and bacteria (aerobic and anaerobic) were negatives after 48 days and 6 days of incubation, respectively. A polymerase chain reaction for Mycobacterium detection was negative.

**Figure 1:**
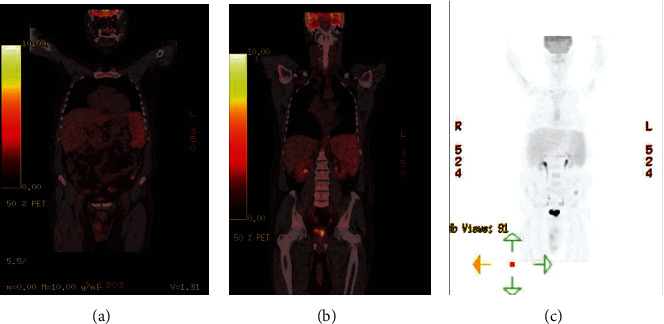
PET-CT scan. Initial PET-CT. (a) Shows the hypermetabolic lesions, which can be seen in the descending colon, and the augmented signaling of the liver without renal compromise is documented. (b, c) PET-CT scans showing nonenhancement of the kidney signal.

A positron emission tomography-computerized tomography (PET-CT) scan was conducted to rule out infectious disease, neoplasms, and inflammatory diseases; it showed a focal hypermetabolic lesion in the colon and an augmented signal in both the spleen and the bone marrow (Figure 1).

**Figure 2:**
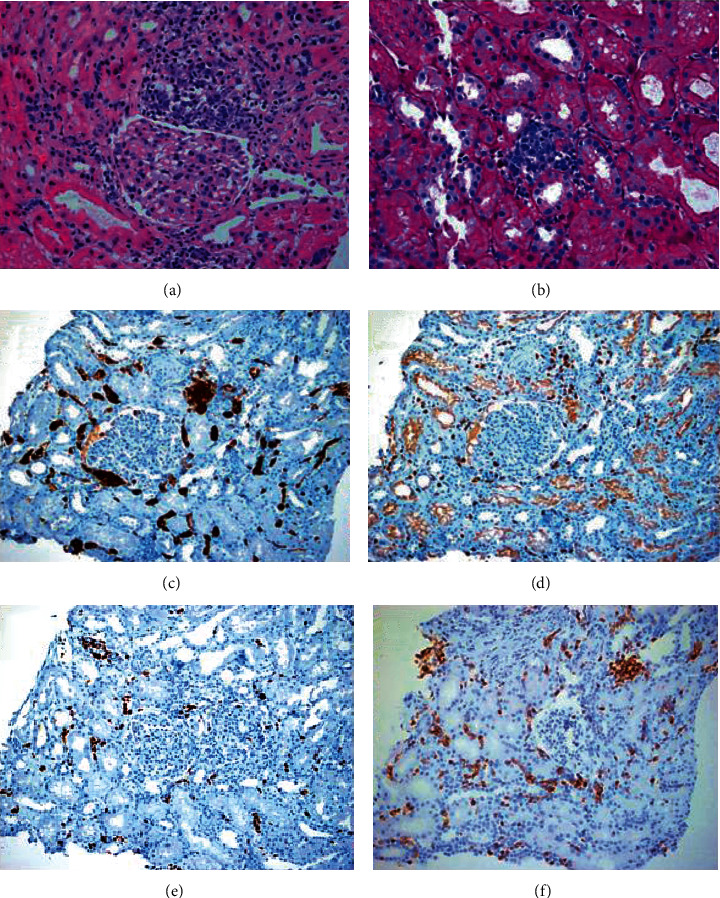
Kidney biopsy. (a) Hematoxylin-eosin staining of the kidney showing the presence of large atypical lymphocytes with an irregular nucleus in the glomerulus and tubules. (b) PAS staining of the kidney showing the presence of lymphocytes in the peritubular capillaries. (c) CD20 staining showing the presence of atypical B-lymphocytes. (d) CD3 staining. (e) KI67 staining showing a proliferation (80%). (f) CD5 staining was positive in this patient and associated with a poor prognosis in IVLBCL.

A colonoscopy found grade 1 internal hemorrhoids and sigmoid diverticulitis with focal edema of the mucosa and small angiodysplasia without signs of bleeding. No tumors or polyps were observed. A biopsy was taken from edematous mucosa. Due to the possibility of insidious diverticulitis, a control CT scan was also ordered. The results of the colon biopsy, alpha-fetoprotein test, CA-19-9 blood test, and carcinoembryonic antigen test were negative for malignancy. The control CT scan showed an enlarged liver along with splenomegaly, and thus, a liver biopsy was performed which showed no alterations. Ten days after admission, the patient developed edematous syndrome along with low serum albumin, elevated serum triglycerides, and elevated urine protein levels. A renal biopsy was performed; it was positive for monoclonal B-lymphocyte (CD20+, CD5+, *BCL2*+ and *BCL6*+, CD3-, GRANZYME-, MUM1-, and CD10- KI67 80%) infiltration inside the peritubular capillaries which is compatible with an intravascular large B-cell lymphoma (Figure 2).

The patient was classified with a poor prognosis and considered high risk according to the Revised International Prognostic Index (R-IPI) due to a compromised extranodal site, an ECOG performance status higher than 2, high levels of lactate dehydrogenase, and an Ann Arbor clinical stage of IV. The karyotype analysis was normal, and the cytogenetic FISH assessment was negative for *BCL2*, *MYC*, and *EBV.*

**Figure 3:**
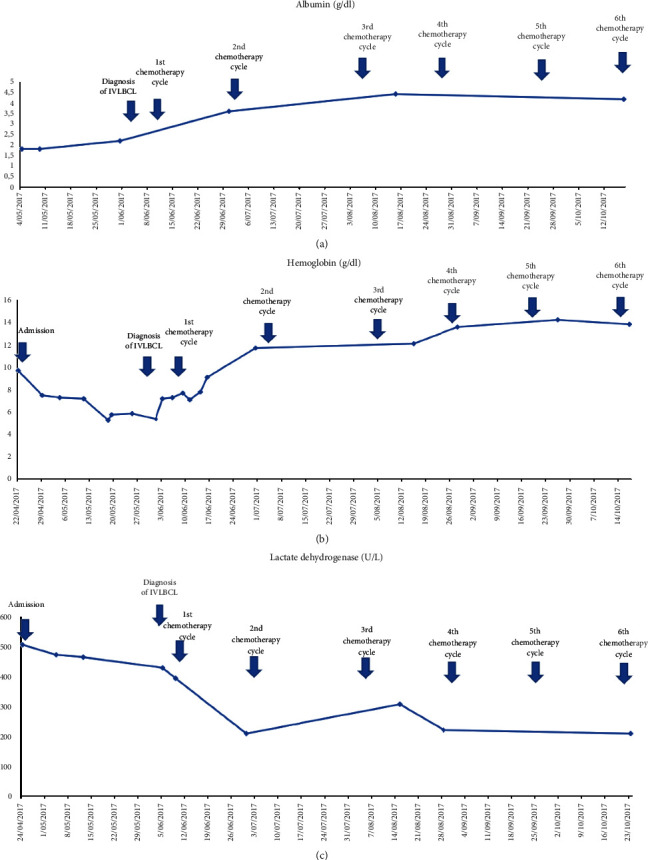
Evolution of the main laboratory parameters. The patient showed improvement in albumin, lactate dehydrogenase, and hemoglobin levels after the chemotherapy treatment. (a) The albumin levels of the patient showed improvement as a result of the treatment. (b) The hemoglobin levels show improvement as a result of the treatment. (c) The lactate dehydrogenase levels decreased as a result of the treatment.

After the diagnosis was made, the patient received 6 cycles of chemotherapy treatment (1 every 21 days) with rituximab, cyclophosphamide, vincristine, doxorubicin, prednisone, intrathecal cytarabine, and methotrexate. This treatment resulted in improvements in his blood test (Figure 3). By the end of the treatment, the patient had achieved complete metabolic response evidenced by the PET-CT scan, normal hemoglobin levels of 14.2 gr/dL, normalized renal function with a creatinine level of 1.01 mg/dL, and regular levels of urine protein. Currently, the patient remains in complete metabolic response after 33 months.

## 3. Discussion

IVLBCL has been commonly characterized as an aggressive disease with a poor overall survival rate, and many diagnoses were determined during postmortem analysis. Even though medical advances have been made in the field of hematological malignancies, intravascular lymphomas continue to be a diagnostic challenge due to the undefined symptoms, variable clinical presentations, and inconclusive imaging studies [8]. Not all patients can be categorized under the three variants of clinical manifestation. Additionally, PET-CT scans of patients with IVLBCL are difficult to analyze due to renal excretion of the radioisotope [6]. This difficulty shows the importance that histopathological studies in patients with compromised organs have in diagnosing and characterizing the disease.

This pathology is considered a rare disease; most of the information about it comes from case reports, small case series, and expert opinions [1]. After conducting a literature review, we found 9 cases of renal IVLBCL [11–19] with a higher presentation in female patients and a median age of presentation of 58. The main clinical manifestations in these cases were fever, renal failure, proteinuria, and edema. In imaging studies, a majority of these patients had enlarged kidneys on CT scans and PET-CT exams that were negative for malignancy. Regarding their histopathology, bone marrow biopsies were normal in almost all the patients, and kidney biopsies showed atypical lymphocytes located primarily in the glomerulus, followed by peritubular and interstitial infiltrates (Table 1). In another study, Törnroth et al. [20] reported 44 cases of bilateral renal lymphoma in 2013. Imaging studies revealed that 40 out of the 44 patients in their study had bilateral enlargement of their kidneys without proteinuria in the nephrotic range. Six patients had bone marrow involvement while 16 had another organ compromised. The mortality rate for these cases was approximately 54.5%.

Our patient presented with nonspecific symptoms and without neurological alterations or skin lesions, which led to extended laboratory testing, a late diagnosis, and the delayed initiation of chemotherapeutic treatment. Notable inconclusive tests, in this case, include a bone marrow biopsy which was negative for neoplasm and hemophagocytic syndrome as well as CT scans and PET-CT scans that did not show any findings regarding the neoplasm. This case presents itself as atypical because the PET-CT scan performed was negative for hypermetabolic lesions in the renal parenchyma. Even upon retroactive revision of the images after the full diagnosis was reached, we were unable to identify suspicious images in the kidneys. This absence of findings comes as a surprise given the current opinion of some experts that positions a PET-CT scan as an important tool for diagnosis and follow-up for IVLBCL, especially in patients with renal compromise [21]. Finally, elevated urine proteins along with the presentation of the edematous syndrome were the sole manifestations that indicated the organ was compromised by the disease.

The introduction of novel agents, such as rituximab, into cancer treatment protocols, has led to a paradigm shift in the treatment of lymphomas, making an early diagnosis and consequent treatment of intravascular lymphoma even more important than ever to secure a better response to therapy [7]. In the small series of cases reviewed in the literature, we found 46% of the subjects were treated with rituximab-based chemotherapy regimens, and all of them reached a complete response with only 1 subject experiencing a relapse of his hematological disease. Also, the mortality rate of IVLBCL has decreased from 54% to 10% [11–19]. It is important to perform, in certain specific cases, a histopathological diagnosis in patients with a fever of unknown origin and/or long duration and organ dysfunction. In this case, the patient did not have any infectious or autoimmune disease or any classical clinical presentation of malignancies. Even though the patient's imaging tests were negative, the presence of hematooncological conditions was determined by renal biopsy.

## Figures and Tables

**Table 1:** Patients with intravascular large B-cell lymphoma.

Case	Age/gender	Year	Clinical manifestation	CT/PET-CT	Bone marrow biopsy	Renal biopsy	Treatment	Outcome
Niitsu et al. [11]	52 (M)	2008	Edema of his lower extremities and mild renal dysfunction.	Computed tomography (CT) revealed markedly enlarged bilateral kidneys but no lymphadenopathy. FDG-PET revealed no abnormal uptake of FDG.	NA	The proliferating cells were phenotypically characterized to be CD3−, CD20+, CD5−, CD10−, BCL2+, BCL6+, and MUM-1+.	R-CHOP	CR

D'Agati et al. [12]	62 (F)	1989	Fever, nausea, vomiting, nephritic syndrome.	NA	NA	MCD with lymphoma cells within glomerular capillaries, interstitial vessel, and interstitium.	Prednisone	Death

Axelsen et al. [13]	60 (F)	1991	Fever, proteinuria, CNS involvement.	NA	NA	Glomerular capillary lumina with large lymphoma cells; CD20+, UCHL-1−, and CD43−.	CAVP	CR

Sekulic et al. [14]	59 (M)	2016	Fever, albuminuria.	PET-CT showed that this lesion had no increased FDG uptake.	Normal	The lymphoma cells were positive for CD20, CD45, CD34, CD31, and immunoglobulin (Ig) M and were negative for cytokeratin, IgG, IgA, and CD56.	R-CHOP	CR

Hasegawa et al. [15]	65 (F)	2015	Renal dysfunction, proteinuria.	CT showed both kidneys were small with a long axis of 9 cm bilaterally.	NA	Atypical large lymphoid cells in the glomerular capillaries were positive for CD20 and CD79a but negative for CD3 and CD10. Lymphocytes in the tubulointerstitium were positive for CD3 but negative for CD20 and CD79a.	R-CHOP	Relapse

Bilgili et al. [16]	56 (F)	2013	Anasarca edema, acute renal failure.	CT showed hepatomegaly with 168 mm size and inguinal lymph nodes.	NA	The immunohistochemical analysis was positive for CD20, factor-8, and leukocyte common antigen (LCA) (CD45) and negative for CD3, CD30, HMB45, S100, CD117, cytokeratin, CK7, CK20, CEA, and EMA.	R-CHOP	CR

Kamalanathan et al. [17]	77 (F)	2013	Renal impairment, proteinuria, lethargy, weight loss.	PET-CT showed diffuse marrow activity but no abnormal renal signal.	NA	Atypical CD20 positive B-lymphocytes filling the capillary lumens of nearly 50% of all glomeruli seen on the biopsy indicating large B-cell lymphoma of nongerminal centre immunophenotype.	R-CHOP	CR

Bai et al. [18]	41 (F)	2011	Fever, bilateral lower limb fatigue.	PET-CT shows foci with high signal intensity in bilateral kidneys, multiple vertebrae, bilateral sacrum, and ilium.	41.5% immature cells of unknown origin.	Atypical lymphoid cells were positive for B-cell markers CD20 and negative for CK, CD3, CD45RO, and CD10. They were positive for large B-cell lymphoma marker mum-1.	CHOP	CR

Kameoka et al. [19]	40 (F)	2006	Proteinuria.	PET-CT revealed no significant uptake of isotopes.	NA	The atypical large lymphoid cells showed positive stainings for leukocyte common antigen CD45, BCL-2, BCL-6, B-cell-associated antigens including CD20, CD79a, and MUM1 and showed negative stainings for CD3, CD5, CD10, or CD56.	R-CHOP	CR

Current case	56 (M)	2016	Fever, headache, dyspnea, arthralgia.	PET-CT showed a focal hypermetabolic lesion in the colon and augmented signal in both spleen and bone marrow.	Normal	Intravascular B lymphoma (CD20+, CD5+, BCL2+ Y BCL6+, CD3-, GRANZYME-, MUM1- Y CD10- KI67 80%).	R-CHOP	CR

R-CHOP: rituximab, cyclophosphamide, doxorubicin, vincristine, and prednisolone; CHOP: cyclophosphamide, doxorubicin, vincristine, and prednisolone; CAVP: cyclophosphamide, doxorubicin, vincristine, and prednisone; CR: complete response; NA: not available.

## References

[1] Rajyaguru D. J., Bhaskar C., Borgert A. J., Smith A., Parsons B. (2017). Intravascular large B-cell lymphoma in the United States (US): a population-based study using Surveillance, Epidemiology, and End Results program and National Cancer Database. *Leukemia & Lymphoma*.

[2] Ponzoni M., Campo E., Nakamura S. (2018). Intravascular large B-cell lymphoma: a chameleon with multiple faces and many masks. *Blood*.

[3] Ponzoni M., Ferreri A. J., Campo E. (2007). Definition, diagnosis, and management of intravascular large B-cell lymphoma: proposals and perspectives from an international consensus meeting. *Journal of Clinical Oncology*.

[4] Ferreri A. J., Campo E., Seymour J. F. (2004). Intravascular lymphoma: clinical presentation, natural history, management and prognostic factors in a series of 38 cases, with special emphasis on the 'cutaneous variant'. *British Journal of Haematology*.

[5] Ferreri A. J., Dognini G. P., Campo E. (2007). Variations in clinical presentation, frequency of hemophagocytosis and clinical behavior of intravascular lymphoma diagnosed in different geographical regions. *Haematologica*.

[6] Desclaux A., Lazaro E., Pinaquy J. B., Yacoub M., Viallard J. F. (2017). Renal intravascular large B-cell lymphoma: a case report and review of the literature. *Internal Medicine*.

[7] Vieites B., Fraga M., Lopez-Presas E., Pintos E., Garcia-Rivero A., Forteza J. (2005). Detection of t(14;18) translocation in a case of intravascular large B-cell lymphoma: a germinal centre cell origin in a subset of these lymphomas?. *Histopathology*.

[8] Yamamoto K., Okamura A., Yakushijin K., Hayashi Y., Matsuoka H., Minami H. (2014). Tandem triplication of the *BCL2* gene in CD5-positive intravascular large B cell lymphoma with bone marrow involvement. *Annals of Hematology*.

[9] Ferreri A. J., Dognini G. P., Govi S. (2008). Can rituximab change the usually dismal prognosis of patients with intravascular large B-cell lymphoma?. *Journal of Clinical Oncology*.

[10] Fonkem E., Lok E., Robison D., Gautam S., Wong E. T. (2014). The natural history of intravascular lymphomatosis. *Cancer Medicine*.

[11] Niitsu N., Okamura D., Takahashi N. (2009). Renal intravascular large B-cell lymphoma with early diagnosis by renal biopsy: a case report and review of the literature. *Leukemia Research*.

[12] D'Agati V., Sablay L. B., Knowles D. M., Walter L. (1989). Angiotropic large cell lymphoma (intravascular malignant lymphomatosis) of the kidney: presentation as minimal change disease. *Human Pathology*.

[13] Axelsen R. A., Laird P. P., Horn M. (1991). Intravascular large cell lymphoma: diagnosis on renal biopsy. *Pathology*.

[14] Sekulic M., Martin S., Lal A., Weins A. (2018). Intravascular large B-cell lymphoma of the kidney. *Kidney International Reports*.

[15] Hasegawa J., Hoshino J., Suwabe T. (2015). Characteristics of intravascular large B-cell lymphoma limited to the glomerular capillaries: a case report. *Case Reports in Nephrology and Dialysis*.

[16] Bilgili S. G., Yılmaz D., Soyoral Y. U., Karadag A. S., Bayram I. (2013). Intravascular large B-cell lymphoma presenting with anasarca-type edema and acute renal failure. *Renal Failure*.

[17] Kamalanathan M., Wright D., Johnston R., Webb A., Kingdon E. (2013). Intravascular large B-cell lymphoma diagnosed at renal biopsy. *Clinical Kidney Journal*.

[18] Bai X., Li X., Wan L., Wang G., Jia N., Geng J. (2011). Intravascular large B-cell lymphoma of the kidney: a case report. *Diagnostic Pathology*.

[19] Kameoka Y., Takahashi N., Komatsuda A. (2009). Kidney-limited intravascular large B cell lymphoma: a distinct variant of IVLBCL?. *International Journal of Hematology*.

[20] Törnroth T., Heiro M., Marcussen N., Franssila K. (2003). Lymphomas diagnosed by percutaneous kidney biopsy. *American Journal of Kidney Diseases*.

[21] Albano D., Laudicella R., Ferro P. (2019). The role of 18F-FDG PET/CT in staging and prognostication of mantle cell lymphoma: an Italian multicentric study. *Cancers*.

